# Ferulic Acid and Salicylic Acid Foliar Treatments Reduce Short-Term Salt Stress in Chinese Cabbage by Increasing Phenolic Compounds Accumulation and Photosynthetic Performance

**DOI:** 10.3390/plants10112346

**Published:** 2021-10-29

**Authors:** Ida Linić, Selma Mlinarić, Lidija Brkljačić, Iva Pavlović, Ana Smolko, Branka Salopek-Sondi

**Affiliations:** 1 Department for Molecular Biology, Ruđer Bošković Institute, 10 000 Zagreb, Croatia; ida.linic@irb.hr (I.L.); iva.pavlovic@irb.hr (I.P.); ana.smolko@irb.hr (A.S.); 2 Department of Agriculture and Nutrition, Institute of Agriculture and Tourism, 52 440 Poreč, Croatia; 3 Department of Biology, Josip Juraj Strossmayer University of Osijek, 31 000 Osijek, Croatia; smlinaric@biologija.unios.hr; 4 Department for Organic Chemistry and Biochemistry, Ruđer Bošković Institute, 10 000 Zagreb, Croatia; lidija.brkljacic@irb.hr

**Keywords:** *Brassica rapa* ssp. *pekinensis*, phenolic compounds, foliar treatment, photosynthetic performance, salicylic acid, ferulic acid, salt stress

## Abstract

Salinity stress is one of the most damaging abiotic stresses to plants, causing disturbances in physiological, biochemical, and metabolic processes. The exogenous application of natural metabolites is a useful strategy to reduce the adverse effects of stress on crops. We investigated the effect of foliar application of salicylic acid (SA) and ferulic acid (FA) (10–100 μM) on short-term salt-stressed (150 mM NaCl, 72 h) Chinese cabbage plants. Subsequently, proline level, photosynthetic performance, phenolic metabolites with special focus on selected phenolic acids (sinapic acid (SiA), FA, SA), flavonoids (quercetin (QUE), kaempferol (KAE)), and antioxidant activity were investigated in salt-stressed and phenolic acid-treated plants compared with the corresponding controls. Salt stress caused a significant increase in SA and proline contents, a decrease in phenolic compounds, antioxidant activity, and photosynthetic performance, especially due to the impairment of PSI function. SA and FA treatments, with a concentration of 10 μM, had attenuated effects on salt-stressed plants, causing a decrease in proline and SA level, and indicating that the plants suffered less metabolic disturbance. Polyphenolic compounds, especially FA, SiA, KAE, and QUE, were increased in FA and SA treatments in salt-stressed plants. Consequently, antioxidant activities were increased, and photosynthetic performances were improved. FA resulted in a better ameliorative effect on salt stress compared to SA.

## 1. Introduction

Global warming and current climate changes are serious threats to agricultural production and biodiversity worldwide [1]. Among abiotic stresses, salt stress is one of the most damaging for plants because it is a complex stress consisting of ionic stress caused by toxic ion concentrations (mainly Na^+^), osmotic stress caused by the associated decreased water uptake, and oxidative stress caused by the increase in reactive oxygen species levels (ROS) [2]. Increased soil salinity is caused by natural processes (such as mineral weathering or by the gradual retreat of an ocean) but also by various human activities mainly related to agricultural practices (especially soil irrigation, fertilization, etc.). It is estimated that about 6% of all land areas are affected by salt, with about 22% of cultivated fields and 33% of irrigated fields used for agriculture. The problem is particularly severe in Mediterranean, semi-arid, and arid areas and is predicted to be more drastic in the future [3].

Salinity stress causes disturbances in physiological, biochemical, and metabolic processes important for plant growth and development. It can alter gene transcription, primary and secondary metabolism, cause an increase in ROS level, affect ion homeostasis, damage photosynthetic apparatus and reduce photosynthetic output, alter membrane permeability, inhibit growth and biomass accumulation, and consequently leads to a significant reduction in crop yield [4]. 

*Brassica* vegetables are among the economically important crops grown worldwide that can be seriously affected by salt stress. All developmental stages of *Brassica* species, from seed germination to seedlings and adult plants, may be threatened by elevated salinity, depending on the natural salt tolerance of the species, duration of stress, and salt concentration [2]. It has been reported that salt tolerance depends on the ploidy of the *Brassica* genome. Accordingly, *Brassica* species that are amphitetraploid, such as *B. juncea*, *B. carinata*, and *B. napus*, are relatively more tolerant to salt stress compared to their diploid parents *B. campestris*, *B. nigra*, and *B. oleracea* [5]. However, there is some variation in salt tolerance within species (amphitetraploid or diploid) and even within cultivars of the same species [6,7]. 

Salt stress response in the *Brassica* species and plants, in general, is a complex trait regulated by many factors (plant hormones, antioxidants, such as polyphenolic compounds, osmoprotectants, etc.) [2,8]. Polyphenols are a diverse group of specialized metabolites that are generally recognized as molecules involved in stress protection in plants, mainly because of their antioxidant activity [9]. It is reported that plants with increased phenolic content under abiotic stress usually show better adaptability to limiting environmental conditions (salinity, drought, temperature, heavy metals, etc.) [10]. 

Selection and breeding of salt-tolerant species and strategies to increase salinity tolerance of sensitive species are of great importance and agronomic interest. In this regard, there are genetically-based strategies, from classical crossing to modern transgene manipulations and genome editing, which are more or less time-consuming methods. In addition to genetic strategies, exogenous application of various agents, such as natural metabolites or synthetic compounds, has been shown to be an excellent way to decrease the adverse effects of salt stress in several important agricultural crops without altering their genome [4]. The exogenous application of numerous natural bioactive molecules (osmoprotectants, plant hormones, antioxidants, etc.) has been found to be useful in neutralizing the deleterious effects of salt stress in various plants [11]. Here we are particularly interested in the application of phenolic acids and their potential mitigating effect on salt stress. The application of SA has been reported to have great agronomic potential in improving the stress response of various agriculturally important crops (barley, maize, sunflower, wheat, bean, strawberry, chamomile etc.) [12]. Several beneficial effects of SA have been reported for plants under salt stress: the alleviation or decrease in photosynthetic pigments and photosynthetic performance, maintenance of membrane integrity, promotion of accumulation of ABA and proline, decrease in lipid peroxidation and membrane permeability, decrease in Na^+^ content and increase in K^+^ concentration, etc. [12].

Aside from SA, a better response of wheat seedlings was obtained in salt stress after treatment with sinapic acid, caffeic acid, ferulic acid, and *p*-coumaric acid [13]. In addition, the application of vanillic acid can decrease the negative effects of salt stress in tomato plants [14], caffeic acid protects cucumber from chilling stress [15], and treatment with ellagic acid accelerated the germination and seedling growth of chickpea under osmotic stress conditions [16]. All these phenolic acids were reported to improve plant antioxidant status by increasing the activities of antioxidant enzymes and the accumulation of non-enzymatic antioxidants. 

Our recent comparative studies on three *Brassica* crops (kale, white cabbage, and Chinese cabbage) showed a positive correlation of phenolic acids and salinity tolerance and clearly identified kale as the most tolerant, then white cabbage as moderately tolerant, and finally Chinese cabbage as the most sensitive species [17]. We showed that the more tolerant kale and white cabbage have significantly higher levels of phenolic acids, especially hydroxycinnamic acids (mainly sinapic acid (SiA), ferulic acid (FA), caffeic acid (CaA), 4-coumaric acid (pCoA)) and suffer less from metabolic disorders under salt stress than the more sensitive Chinese cabbage. Therefore, our aim was to investigate if the foliar application of selected phenolic acids may diminish the adverse effect of short-term salt stress (72 h) in Chinese cabbage. For this purpose, solutions of salicylic acid (representative of hydroxybenzoic acids and plant stress hormone) and ferulic acid (representative of hydroxycinnamic acids) were sprayed at concentrations of 10–100 μM onto leaves of one-month-old Chinese cabbages treated with 150 mM NaCl. Stress levels, photosynthetic performance, phenolic metabolites, and antioxidant activity were then examined under the control and salt stress conditions (150 mM).

## 2. Results

One-month-old plantlets of Chinese cabbage were foliar-treated by a spray applicator with FA and SA in the concentration range of 10–100 µM, approximately 0.5 mL per plant per treatment, and their effects were investigated under NaCl stress conditions (150 mM, in the time period of 72 h) compared to untreated controls. Short-time salinity stress caused visible plant dehydration that was less prominent in plants treated with phenolic acids. Changes in growth parameters were not observable under the short experimental period (72 h). 

To access biochemical changes, we measured proline as the reliable stress marker, groups of polyphenolic compounds (total polyphenols, total phenolic acids, total flavonoids and total flavanols), as well as the most abundant phenolic compounds well known as powerful antioxidants in Brassicaceae (ferulic acid (FA), sinapic acid (SiA), salicylic acid (SA), kaempferol (KAE), and quercetin (QUE) and antioxidant activities. Furthermore, selected photosynthetic parameters were screened by chlorophyll *a* fluorescence.

### 2.1. Proline Level

**Figure 1:**
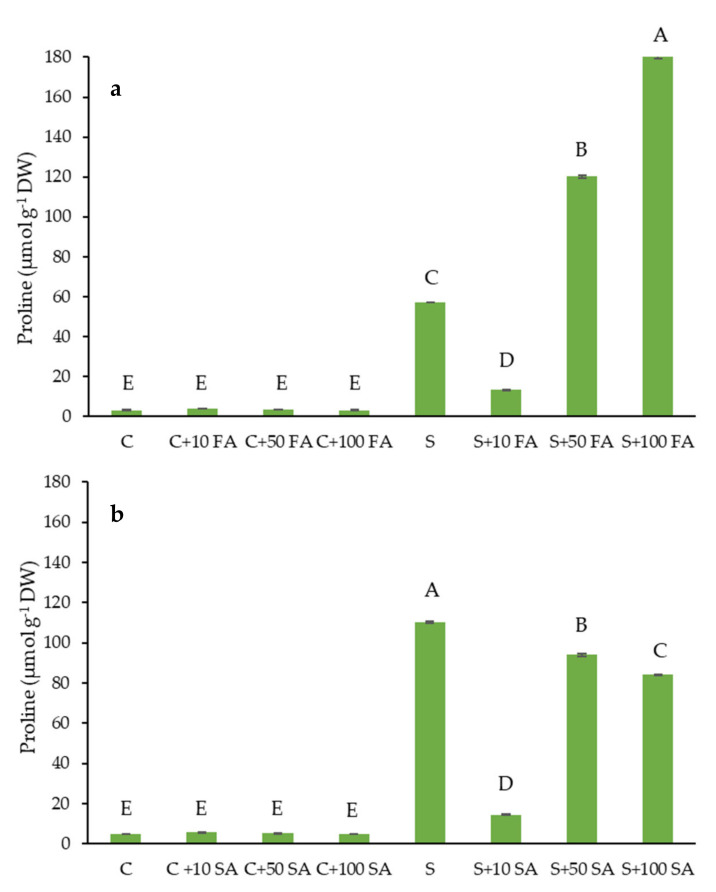
Effect of foliar treatments of *B. rapa* plants with: (**a**) ferulic acid (FA) and (**b**) salicylic acid (SA) at concentrations 10, 50, and 100 µM on proline content under salt treatment (S, 150 mM NaCl) compared to untreated control (C), for 72 h. Data are mean ± sd (n = 3). Proline is expressed as μmol per g dry weight (μmol g^−1^ DW). Different letters in each plot represent statistically significant differences at *p* < 0.05.

To assess the plant stress response to NaCl-treated plants and identify the effect of phenolic acids (FA and SA) on the stressed plants, we analyzed proline as a salt stress marker in eight sets of experimental plants (Figure 1).

Phenolic acid treatments had no significant effect on proline content in plants grown under non-saline conditions compared to untreated controls. This suggests that phenolic acids per se did not cause stress in treated plants. Under salt stress, proline contents increased significantly (18 and 23-fold in FA and SA experimental sets, respectively) compared to the unstressed controls (Figure 1). Treatments with both phenolic acids (FA and SA) at a concentration of 10 μM caused a significant reduction in proline content. Proline content was reduced about five-fold in foliar treatments compared with content in salt-stressed, untreated plants. The application of higher concentrations (50 and 100 μM) of both phenolic acids resulted in increased proline content. The increase in proline was more pronounced in FA than in SA treatments (50 and 100 μM) compared to 10 μM concentration and untreated plants.

### 2.2. Chlorophyll a Fluorescence Parameters

**Figure 2:**
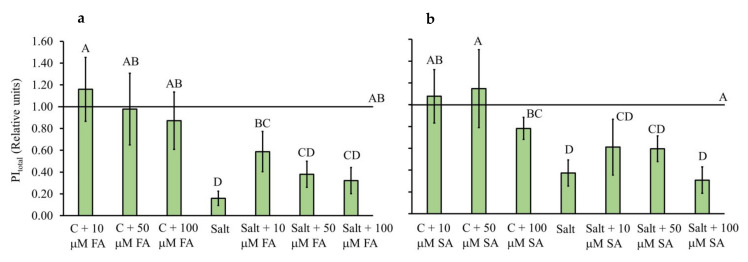
Effect of foliar treatments of *B. rapa* plants with ferulic acid (FA) (**a**) and salicylic acid (SA) (**b**) in concentrations of 10, 50, and 100 µM on the overall photosynthetic performance index (PI_total_) due to salt treatment (150 mM) for 72 h. All values are normalized to untreated control plants (C), presented with the line at value 1. Data are mean ± sd (n = 6). Different letters in each plot represent statistically significant differences at *p* < 0.05.

We investigated the effect of FA and SA treatments on photosynthetic parameters of salt-stressed Chinese cabbage plants by measuring fast chlorophyll *a* fluorescence kinetics. Figure 2 shows the total photosynthetic performance index (PI_total_) (see description of parameters in Table 1) in FA and SA-treated plants (10–100 µM) under saline conditions (150 mM NaCl) compared to untreated plants grown under control conditions. PI_total_ is the most sensitive and the most informative photosynthetic parameter, which indicates the overall functional activity of both photosystems (PSII and PSI) and the intersystem electron transport chain [18,19,20].

As can be seen, PI_total_ was not affected in the control plants treated with 10 and 50 µM SA and FA, although a higher concentration (100 µM) showed a tendency to reduce PI_total_, especially at SA. 

Salt stress significantly decreased PI_total_ (up to 80%) in Chinese cabbage leaves. Treatments of salt-stressed plants with 10 µM FA and SA showed a tendency to improve photosynthetic performance by 6- and 1.5-fold, respectively, with a statistically significant improvement, observed only in the treatment with 10 µM FA compared to salt-stressed plants. 

**Figure 3:**
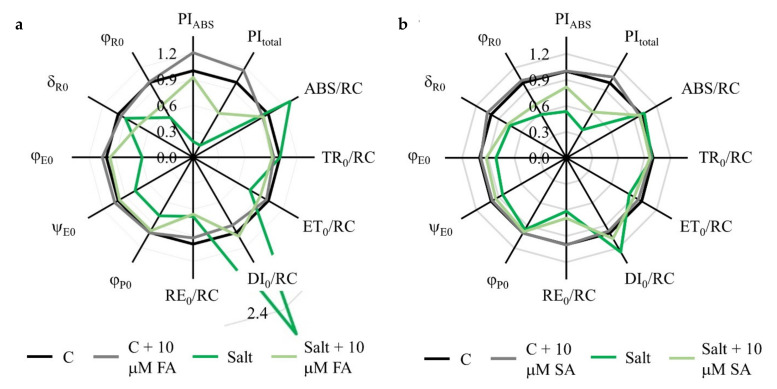
Effect of foliar treatments of *B. rapa* plants with ferulic acid (FA) (**a**) and salicylic acid (SA) (**b**) at concentrations of 10 µM on the photosynthetic parameters due to salt treatment (150 mM) for 72 h. All values are expressed in relation to the untreated control (C), presented with the line at value 1. Abbreviations and descriptions of parameters are given in Table 1. Original data are shown in Table S1.

Since the screening of applied concentrations of phenolic acids selected the 10 µM concentration as one with a positive effect on PI_total_, we further investigated the photosynthetic mechanisms in samples of foliar treated with this favorable concentration of SA and FA. All investigated parameters are described in Table 1. Spider plots (Figure 3) show normalized values of calculated biophysical parameters in salt-stressed plants and salt-stressed plants treated with phenolic acids relative to unstressed controls.

Although the experimental conditions were the same for the two sets of experiments and the trends in the parameters were the same, slight differences were observed in the salt-stressed plants. 

**Figure 4:**
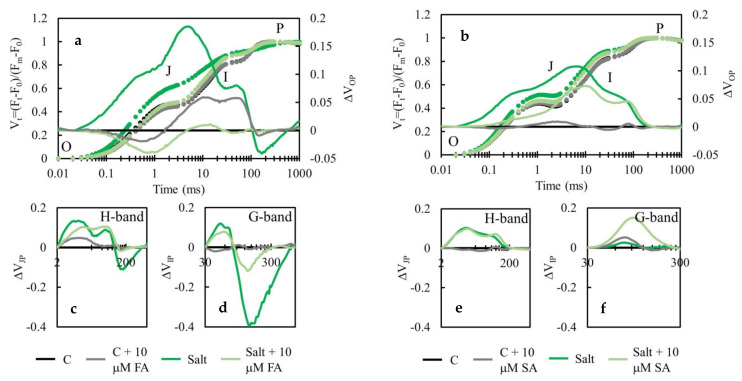
Effect of foliar treatments with ferulic acid (FA; (**a**,**c**,**d**)) and salicylic acid (SA; (**b**,**e**,**f**)) at a concentration of 10 µM on the chlorophyll a fluorescence transient curves due to salt treatment (150 mM) for 72 h compared to the untreated control (C). Kinetics data were normalized between O-P (**a**,**b**), J-P (H-band; (**c**,**e**)), and I-P (G-band; (**d**,**f**)) steps and plotted as difference kinetics ΔVt in corresponding time ranges. Each curve represents the average kinetics of six replicates per treatment. Typical O-J-I-P steps are marked in the relative variable fluorescence transient, ΔVt (**a**,**b**).

Photosynthetic performance parameters PI_ABS_, and consequently PI_total_, showed significant decreases in salt-stressed plants compared to the controls (PI_ABS_ is decreased in 19% while PI_total_ in 16% of the untreated controls). Photosynthetic performance indexes did not suffer such a significant decrease in salt-stressed plants treated with 10 µM FA and SA; PI_ABS_ and PI_total_ were 92% and 61% of the corresponding control values, respectively. Processes in reactive centers (RC) were differentially affected by salinity stress; while trapping flux per active RC (TR_0_/RC) and electron transport flux per active RC (ET_0_/RC) were not affected drastically, absorption flux per active RC (ABS/RC) and dissipation flux per active RC (DI_0_/RC) were significantly increased upon salinity stress. Mitigating effects of SA and FA treatments were obvious, and maintained ABS/RC and DI_0_/RC values on the levels of untreated controls. Moreover, electron flux reducing end electron acceptors at the PSI acceptor side, per RC (RE_0_/RC) was significantly decreased in salt-stressed plants (about 40%), and phenolic acid treatments did not show a positive effect on this parameter. The maximum quantum yield of PSII (φ_P0_) was about 22% lower in salt-stressed plants compared to the control but may be protected with FA and SA treatments. The quantum yield for reduction of end electron acceptors at the PSI (φ_R0_) was up to 40% lower in salt-treated plants compared to corresponding controls and may be significantly protected by phenolic acid treatments. The quantum yield for electron transport (φ_E0_) was also decreased by salt up to 40%, and the negative effect of salt on this parameter may be alleviated by phenolic acids treatments. These data implicate that PSI is more sensitive to salinity stress than PSII. The influence of salt stress on PSI is even more clearly presented in O-P normalized curves (Figure 4). The O-P curves indicated differences in PSII and PSI functionality between control and salt-stressed plants, as well as salt-stressed plants treated with 10 µM FA and SA. They demonstrated that the fluorescence intensity at defined transient steps was successively increased in salt stress conditions compared to the untreated controls, while FA and SA treatments of salt-stressed plants maintained the fluorescent intensities at the level of controls. This finding confirms a protective role of applied phenolic acids for photosynthetic apparatus. 

H- and G- bands were the highest for salt-treated plants, while treatment with FA showed a protective role against salinity on the photosynthetic apparatus and decreased specific bands.

### 2.3. Phenolic Compounds and Antioxidative Activities

Phenolic compounds are a diverse group of specialized metabolites generally involved in plant protection against abiotic stresses [9]. Table 2 shows the results of polyphenolic groups’ contents and antioxidant activities in an experimental setup with FA treatment, while Table 3 presents data in an experimental setup with SA treatment. Under our experimental conditions, the untreated controls of *B. rapa* plants contained total phenols of 28–33 mg of gallic acid per g of dry weight (GAE g^−1^ DW), total phenolic acids of 5–7 mg of caffeic acid per g of dry weight (CAE g^−1^ DW), total flavonoids of 6–8 mg of catechin per g of dry weight (CE g^−1^ DW), and total flavanols of 58–66 µg CE g^−1^ DW (Table 2 and Table 3). Antioxidant activities evaluated by ferric reducing/antioxidant power assay (FRAP assay) and 2,2-Diphenyl-1-picrylhydrazyl assay (DPPH assay) were 32–42 µmol Trolox per g of dry weight (TE g^−1^ DW) and 41–57 µmol Fe^2+^ g^−1^ DW, respectively (Table 2 and Table 3). Salt stress significantly decreased the content of all groups of phenolic compounds by 20–35%, as well as antioxidant activity compared to the untreated control (Table 2 and Table 3). 

Treatments of unstressed and salt-stressed plants with FA (Table 2) and SA (Table 3) resulted in changes in phenolic compound content and antioxidant activities. Treatment of controls with FA at a concentration of 10 μM significantly increased total phenolics and total flavanols, as well as antioxidant activity, as determined by FRAP (by 7%, 13%, and 8%, respectively, compared with controls) (Table 2). Higher concentrations of FA showed an inhibitory effect on the accumulation of polyphenols (except flavanols) in unstressed plants. FA treatments showed some positive effects on salt-stressed plants. FA at a concentration of 10 μM significantly increased all groups of phenolic compounds (13–32%) during salt stress compared to untreated salt-stressed plants. The antioxidant capacity of plants treated with 10 μM FA was also significantly increased (approximately 30% compared to salt-stressed plants). SA showed a more positive effect and increased the accumulation of most specialized metabolites and antioxidant activity at all applied concentrations (10, 50, and 100 μM) in unstressed plants (Table 3). Thus, 100 μM SA increased total phenolics, phenolic acids, flavonoids, and antioxidant activity, as measured by DPPH and FRAP, by 9%, 32%, 21%, 10%, and 9%, respectively, compared with controls, whereas flavanols were decreased by 18% compared with controls. SA was less effective than FA under salt stress conditions and caused an improvement in polyphenol accumulation by about 10–26% at concentrations of 50 and 100 μM. Antioxidant activity was improved by up to 20% in salt-stressed plants by SA treatments (100 μM).

Since cabbage plants are known to be rich in the phenolic acids FA, SiA, and SA [17], and the flavonoids, subclass flavonols, kaemferol and quercetin [21], we analyzed these metabolites in detail by LC-MS/MS in *B. rapa* leaves after salt stress and treatments with FA and SA at a 10 μM concentration. Individual metabolites were identified based on retention time (RT) and specific multiple reaction monitoring (MRM) transitions (Table S2, Figures S1 and S2). 

**Figure 5:**
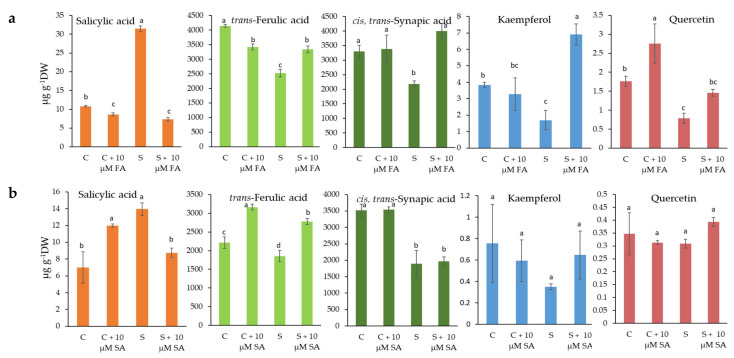
Effect of foliar treatments of *B. rapa* with ferulic acid (FA) (**a**), and salicylic acid (SA) (**b**)) at a concentration of 10 µM on the selected phenolic compounds (Salicylic acid (SA), *trans*-Ferulic acid (*trans*-FA, *cis*, *trans*-Synapic acid (SiA), Kaempferol (KAE), and Quercetin (QUE)) measured by LC-MS/MS due to salt treatment (S, 150 mM NaCl) for 72 h. Data are mean ± sd (n = 3). Different letters represent statistically significant differences for each individual specialized metabolite (*p* < 0.05).

Changes in the content of selected metabolites caused by salinity stress and at FA and SA foliar treatments are shown in Figure 5. As can be seen, the content of FA and SiA were up to 4.1 and 3.5 mg g^−1^ DW, respectively, in the untreated controls, while the concentrations of SA, KAE, and QUE were up to 10.8, 3.8, and 1.7 µg g^−1^ DW.

Salinity stress caused a reduction in all metabolites studied except SA compared to the untreated control. Briefly, the phenolic acids *trans*-FA and SiA were reduced by up to 39% and 46%, respectively. The flavonols KAE and QUE were both reduced by salt stress by up to 56%. On the other hand, SA was significantly increased by salt stress (up to 2.9-fold compared to the untreated control). 

Foliar treatments with FA and SA had a positive effect on stressed plants and caused a significant decrease in SA (2.2- and 1.6-fold, respectively), while other metabolites increased compared to salt-stressed plants. Thus, SA leaf treatment increased *trans*-FA, KAE, and QUE (1.5-, 1.9-, and 1.3-fold, respectively), while it did not affect SiA content. Moreover, FA foliar treatment increased the content of *trans*-FA, SiA, KAE and QUE (1.3-, 1.8-, 4.1-, and 1.9-fold, respectively) significantly, compared to salt-stressed plants.

**Figure 6:**
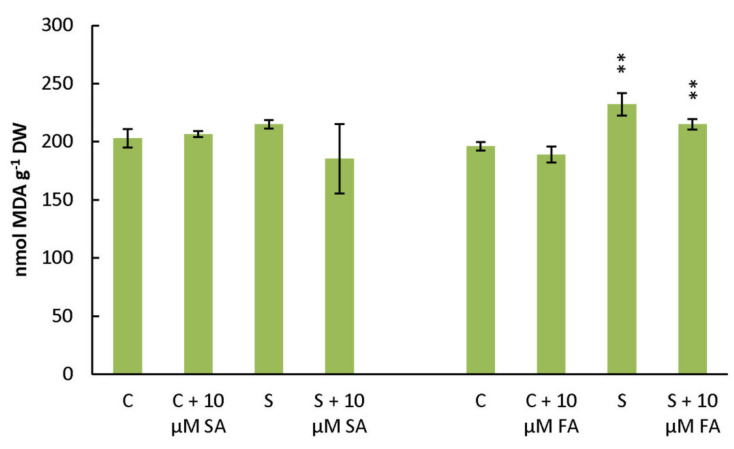
Effect of foliar treatments of *B. rapa* with phenolic acids (SA and FA, at a concentration of 10 µM) on the lipid peroxidation measured by the malonaldehyde (MDA) method due to salt treatment (150 mM NaCl) for 72 h. S, salt treatment; C, control; SA, salicylic acid; FA, ferulic acid. Asterisks indicate a significant difference in the measured parameters in controls compared to treatments in a Student’s *t*-test (** correspond to *p*-values of 0.001 < *p* < 0.01).

To evaluate oxidative stress upon salt stress and phenolic acid treatments, we measured lipid peroxidation and expressed the content of malonaldehyde (MDA). As can be seen in Figure 6, lipid peroxidation levels were increased significantly in salt stress compared to the corresponding controls. Phenolic acid (SA and FA) treatments in salt stress showed the tendency to lower lipid peroxidation; thus, the MDA level was lower in salt-stressed plants treated with phenolic acids compared to salt-stressed plants.

## 3. Discussion

Many species and varieties of Brassica crops, including Chinese cabbage, are germinated and raised to the seedling stage under favorable growing conditions (greenhouse or growth chamber), and then transplanted to the field under environmental conditions. In this early stage, transplanted plantlets are often exposed to unpleasant conditions and may suffer from increased soil salinity. Foliar treatment of salt-sensitive plants with compounds that are at least partly responsible for tolerance in salt-tolerant species is one of the strategies to mitigate the negative effects of increased soil salinity on the plants. Based on our previous studies, FA and SA might be part of the salt response mechanisms in salt-tolerant *Brassica* species (kale) compared to salt-sensitive ones (Chinese cabbage) [17]. Therefore, we investigated here whether foliar application of Chinese cabbage with FA and SA can reduce the effects of short-term salt stress and harden the plants during this sensitive growth period. One-month-old plantlets were subjected to short-term salt stress (150 mM NaCl, for 72 h) and treated with FA and SA at a concentration of 10–100 μM. In our previous publication, we reported short-term salinity responses of three Brassicas, including Chinese cabbage, on the physiological and biochemical levels [7]. Based on our published data [7,17], we decided to evaluate the effects of phenolic acid treatments by measuring only selected markers, such as proline, chlorophyll *a* fluorescence parameters, major groups of phenolic compounds (total polyphenols, total phenolic acids, total flavonoids, total flavanols), antioxidant activity, and selected phenolic compounds (FA, SiA, SA, KAE, QUE), which are known to be major antioxidants in Brassicaceae [17,21,22].

### 3.1. Attenuating Effects of FA and SA on Short-Term Salinity Stressed Plants: Stress Markers

Proline has been confirmed as a reliable stress marker under elevated salinity conditions, suggesting a positive correlation between proline accumulation and plant stress [23,24]. The proline level was 18–23 fold increased after 72 h of salt-stress in Chinese cabbage leaves (Figure 1) compared to the unstressed controls that are in agreement with our previously published data [7]. Proline can act as an osmoprotectant, but it is also known as a metal chelator, an antioxidant defense molecule, and a signaling molecule [24]. Phenolic acids treatments at a concentration of 10 μM decreased the level of proline in salt-stressed plants, suggesting that 10 μM foliar treatments with FA and SA diminish the stress intensity caused by salt in Chinese cabbage plants. This is in agreement with previous research that confirms a positive correlation of the content of proline and the stress intensity in Brassicas [7,25], showing that proline content was increasing in a dose-dependent manner with the intensity of salinity stress in different Brassica crops. The application of higher concentrations of FA (50 and 100 μM) in combination with salinity stress resulted in increased proline content compared to salt-stressed untreated plants. This may suggest that higher concentrations of applied FA in combination with increased salinity caused additional stress for Chinese cabbage plants. It may be due to the pro-oxidant activity of FA at higher concentrations, as already reported [26]. The correlation of proline content, applied FA, and stress response depends on plant species, type of stress, and stress duration. Thus, it was shown that pretreatment with FA increased proline content in cucumber leaves [27] and in blueberries (*Vaccinium corymbosum* L.) [28] and participated in protection from desiccation stress and heat stress, respectively. The application of higher concentrations of SA (50 and 100 μM), in combination with salinity stress, caused a decrease of proline content compared to salt-stressed untreated plants, although they are not as effective as the lowest applied concentration (10 μM).

The correlation between proline accumulation and stress tolerance is controversial, and it is still a matter of debate. In some plant species, high proline accumulation is linked to increased stress tolerance, while in others, it is a sign of stress sensitivity [23,24]. Different correlations between proline and stress are also reported in Brassicaceae. Comparative research between Chinese cabbage, white cabbage, and kale under salinity [7] and drought [29] stress suggest that higher proline content is linked to more sensitive species/varieties. Furthermore, comparative evaluation of different varieties of kale (*Brassica oleracea* var. *acephala*) under osmotic stress showed a higher accumulation of proline in more sensitive varieties (unpublished data). However, Mohamed et al. [30] showed that proline in rapeseed cultivars subjected to salt stress correlated positively with salt tolerance. Hayat et al. [25] also showed a positive correlation between proline accumulation and salt tolerance in a comparative study of different *B. juncea* varieties. Thus, proline accumulation and its correlation with salinity and osmotic stress tolerance seem to be species or even variety-specific in Brassicaceae.

Another stress marker is salicylic acid (SA), which was significantly increased by salt stress (up to 2.9-fold compared to the untreated control) (Figure 5). It is not surprising, since SA acts as a stress hormone and increased levels of SA have already been documented in a number of plants, including Brassicaceae, under stress conditions [7,12]. As described by Khan et al. [31], salicylic acid can regulate various plant metabolic processes and modulate the production of osmolytes and specific metabolites. The exogenous application of SA, and especially FA, lowered the level of endogenous SA, as well as proline level, as markers of stress status under our experimental conditions.

### 3.2. FA and SA Treatments Stabilize Photosynthetic Parameters under Salinity Stress

Photosynthesis has been shown to be negatively affected by salt stress in *Brassica* species depending on natural salt tolerance [7,25,30,32]. The total photosynthetic performance index (PI_total_) is the most sensitive and the most informative photosynthetic parameter, which indicates the overall functional activity of both photosystems (PSII and PSI) and the intersystem electron transport chain [18,19,20]. Short-term salt stress significantly decreased PI_total_ (up to 80%) in Chinese cabbage leaves compared to the unstressed controls. Moreover, parameters, such as RE_0_/RC, φ_R0_ and φ_E0_, were up to 40% lower in salt-treated plants compared to corresponding controls, while φ_P0_ was about 22% lower in salt-stressed plants compared to the unstressed control. Similarly, Dąbrowski et al. [20] reported that the diminution of PSII activity and impairment of PSI function significantly decreased the total performance index in ryegrass. Oukarroum et al. [33] detected salt-induced inhibition of both PSII and PSI electron transport activities in duckweed. 

PI_total_ was not affected in control plants treated with 10 and 50 µM SA and FA, although a higher concentration (100 µM) showed a tendency to reduce PI_total_, especially for SA (Figure 2). This is in agreement with published data reporting that higher SA concentrations (1.0 mM) have an inhibitory effect on photosynthesis and overall growth performance in *B. juncea* cultivars [34]. Moreover, a high concentration of FA (1.5 mM) inhibited photosynthesis and can be used as a bioherbicide [35].

Under the short-term salt stress, the same concentration of FA and SA (10 μM) that lower the proline and SA level in salt-treated plants toward control level showed a positive effect on photosynthetic parameters measured by chlorophyll *a* fluorescence (Figure 3). Treatments of salt-stressed plants with 10 µM FA and SA showed a tendency to improve PI_total_ by 6- and 1.5-fold, respectively. Furthermore, the negative effect of salt on the maximum quantum yield of PSII (φ_P0_), quantum yield for reduction of end electron acceptors at the PSI (φ_R0_), and quantum yield for electron transport (φ_E0_) were alleviated by phenolic acid treatments. The O-P curves demonstrated that the fluorescence intensity at defined transient steps was successively increased in salt stress conditions compared to the untreated controls, while FA and SA treatments of salt-stressed plants maintained the fluorescent intensities at the level of controls (Figure 4). This finding confirms a protective role of applied phenolic acids for photosynthetic apparatuses. Furthermore, FA treatment showed a protective role against salinity on the photosynthetic apparatus and lower specific H- and G-bands due to the possible generation of cyclic electron flow around PSI [36]. However, SA treatment revealed an almost identical amplitude of the H-band in both salt-treated plants, while the G-band in salt-stressed plants showed drastically lower amplitude compared to the SA treated ones. In some cases, a smaller amplitude could be the result of a stronger limitation on PQH_2_ reoxidation due to the slowdown of electrons from PSII (lower φ_E0_ and ψ_E0_) [37].

The protective role of SA to photosynthetic apparatuses has been reported earlier for a number of species [12]. In salt-treated tomato plants, the effective quantum yield (ϕPSII) and the photochemical quenching coefficient (qP), which declined significantly under salt stress, were partially restored in SA hardened plants. Similarly, the net CO_2_ fixation rate was higher in SA-treated tomato than in the salt-treated control [38]. Salinity also reduced photosynthetic efficiency by inhibiting chlorophyll synthesis, nitrate reductase activity, chlorophyll fluorescence, stomatal conductance, net photosynthetic, and transpiration rates in Ethiopian mustard (*Brassica carinata*), and SA application alleviated the adverse effects of salinity and improved the performance of the photosynthetic parameters [39]. 

To our knowledge, the effect of FA on the photosynthetic apparatus has not been studied to date. In a recent publication, it was shown that a high concentration of FA (1.5 mM) inhibits photosynthesis and can be used as a bioherbicide [35], although there are no reports on the effects of lower concentrations of FA on the photosynthetic apparatus. However, it has been shown that plants with higher levels of endogenous FA showed better photosynthetic performance under stress conditions [40]. Up-regulations of 4-hydroxycinnamic acid and ferulic acid were consistent with photosynthetic performance in the early stages of drought in rice, suggesting that 4-hydroxycinnamic acid and ferulic acid are considered key metabolites for drought tolerance in rice [41]. *Brassica* cultivars with a higher content of FA [17] showed better photosynthetic performance under salinity stress [7]. These data suggest a positive correlation between FA and photosynthetic performance depending on concentration, which is confirmed by our results.

### 3.3. FA and SA Treatments Increase an Accumulation of Phenolic Antioxidants and Antioxidant Activity under Stress Conditions 

Metabolic disorder in salt-stressed plants was notable in polyphenolic content and consequently in antioxidant activity. As can be seen in Table 2 and Table 3, salt stress significantly decreased the content of all groups of phenolic compounds by 20–35%, as well as antioxidant activity compared to the untreated control. Furthermore, salinity stress caused a reduction in the selected metabolites studied; *trans*-FA and SiA were reduced by up to 39% and 46%, respectively, the flavonoids KAE and QUE were both reduced by salt stress by up to 56%. Only endogenous SA that acts as a stress hormone and presents one of the stress markers was increased up to 2.9-fold, as we have already discussed above.

It is evident that the treatments of SA and especially FA increased the content of potent phenolic antioxidants (phenolic acids and flavonoids) under stress conditions in the leaves of *B. rapa*, which is consistent with the increased antioxidant activities compared with salt-stressed untreated plants (Table 2 and Table 3). This is in agreement with the level of lipid peroxidation (Figure 6). Salinity stress caused an increase of lipid peroxidation (MDA content) compared to the corresponding controls, while phenolic acid treatments showed the tendency of decreasing the MDA content. In our experimental design, FA appears to be a stronger stimulator of polyphenols accumulation and antioxidant activity than SA. The treatment of salt-stressed plants with 10 μM FA accumulated total polyphenols, total flavanols, and antioxidant activity on the level above that measured in the untreated and unstressed control. This may be explained by the fact that hydroxycinnamic acids (FA), compared with the corresponding hydroxybenzoic acids (SA), exhibit higher antioxidant activity, which has been attributed to the greater hydrogen-donating and radical-stabilizing ability of the CH=CH-COOH group [9].

Higher levels of phenolic compounds and antioxidant activities under stress conditions, after treatments with FA and SA, correlate positively with the improvement of the photosynthetic parameters, the decrease of stress markers (proline and salicylic acid), and decrease of lipid peroxidation level in *B. rapa* plants. The phenylpropanoid biosynthetic pathway has been shown to be activated under abiotic stress conditions in some plants, leading to the accumulation of several phenolic compounds that are widely recognized as protective biomolecules [10]. A positive correlation between increasing salt tolerance and increasing levels of phenolic compounds has been observed in many halophytes (salt-tolerant species) [42]. In addition, salt tolerance has also been reported to correlate with levels of specialized metabolites, including phenolic compounds, in different plant species and cultivars [9]. Transgenic tobacco plants (AroG plants) that had significantly higher levels of phenolic metabolites showed improved tolerance to salt stress [43]. Soil salinity increased the concentrations of leaf phenolics, including chlorogenic acid, in honeysuckle as a mechanism of acclimation to salt stress [44]. Martinez et al. [45] reported that cinnamic acid, *p*-coumaric acid, and p-coumaryl CoA, in addition to flavonols, were several times higher in tomato due to salinity, heat, and combined stress (heat + salinity) compared to control plants. Furthermore, endogenous ferulic acid and *p*-coumaric acid are thought to be involved in the response mechanism to salt stress in rice [46]. Low salinity also increased phenolic compounds at a certain level in various *Brassica* species [47,48].

The exogenous application of phenolic compounds to improve salinity response has been reported mainly through SA as phenolic acid but also as a plant stress hormone [12]. However, the effect of SA depends on the concentration of the applied SA, the plant species, and the developmental stage of the plants. In general, low concentrations of applied SA can improve abiotic stress tolerance, while high concentrations induce oxidative stress, resulting in increased sensitivity to abiotic stress [12]. As described by Khan et al. [31], salicylic acid can regulate various plant metabolic processes, modulate the production of osmolytes and specific metabolites, which is consistent with our results.

Less information is available for FA effects on phenolic compounds and antioxidant activity, as well as stress tolerance. It was shown that in addition to SA, increased salinity tolerance of wheat seedlings was also obtained after treatment with sinapic, caffeic, ferulic, and *p*-coumaric acids [13]. The authors reported that the exogenous application of phenolic acids reduced electrolyte leakage in NaCl-stressed seedlings, decreased H_2_O_2_ and malondialdehyde levels, and activated antioxidant enzymes. Pretreatment with FA protected cucumbers from desiccation stress by reducing lipid peroxidation due to the activation of antioxidant enzymes and by increasing proline and soluble sugar content in leaves [27]. Pretreatment with FA alleviated heat stress in blueberries (*Vaccinium corymbosum*) by increasing the endogenous FA content, which is consistent with our results, and decreasing the accumulation of reactive oxygen species, as well as increasing the proline and soluble sugar content [28].

In addition to phenolic acids, flavonoids are also potent antioxidants capable of inhibiting the formation of ROS and also quenching ROS once formed [9,49]. There are not many reports on the direct involvement of quercetin or kaempferol in salinity stress response. The treatment of tomato with quercetin under salinity resulted in the alleviation of ionic, osmotic, and oxidative stress [50]. Kaempferol, quercetin-3-rutenoside (rutin), and dihydrokaempferol have been reported to accumulate as a stress response under heat and the combination of salt and heat stress, along with increased levels of gene transcripts in the phenylpropanoid pathway, are responsible for their synthesis [45]. Quercetin and kaempferol have been reported to mediate prolonged abiotic stress in peanut (*Arachis hypogaea*) [51].

Treatments with SA and methyl-salicylate (MeSA) were reported to enhance the activity of phenylalanine ammonia-lyase (PAL, a key enzyme in the phenylpropanoid biosynthesis pathway) and upregulate the expression of genes of the phenylpropanoid pathway (such as PAL, C4H, 4CL, CHS, CHI, F3H, DFR, ANS, and UFGT) [31,52]. Under our experimental conditions, the accumulation of FA, KAE, and QUE in salt-stressed plants upon SA treatment suggest that genes involved in their biosynthesis were upregulated by exogenously added SA. There is a lack of information on the FA mode of action upon foliar treatments. Since FA, SiA, KAE, and QUE were increased in salt-stressed plants upon FA treatment; we may speculate that the applied FA stimulated gene expressions of the phenylpropanoid pathway. It is interesting that the non-stressed control showed a lower level of FA upon treatment compared to untreated controls. It may be due to the inhibition of endogenous FA biosynthesis by exogenously applied FA. The mode of action of foliar-applied FA on salt-stressed plants is still unknown and needs to be investigated in the future.

### 3.4. Conclusions

Based on our results, we may conclude that foliar treatments of salt-stressed plants with SA and FA (especially 10 μM concentration) showed a positive effect on salt-stressed plants by attenuating the stress effects. In short, phenolic acid treatments decreased proline and endogenous SA content compared to untreated salt-stressed plants. In addition, the content of phenolic compounds was increased. In particular, an increase in endogenous phenolic acids FA and SiA and flavonoids QUE and KAE was observed. Consequently, antioxidant activity was increased in stressed plants by the SA and FA treatments. In addition, photosynthetic performance was improved. Photosynthetic parameters, such as PI_ABS_, PI_total_, φ_P0_, φ_E0_, ψ_E0_, φ_R0_, and DI_0_/RC, which were significantly disturbed under salt stress, were protected by phenolic acid treatments. FA resulted in a better ameliorative effect on salt stress compared to SA, probably due to a better antioxidant capacity. Treatments of plants with FA and SA can be used to help plants cope with salinity stress, at least during the sensitive period of adaptation of the plantlets after transplantation to the field. However, the mechanisms of action of phenolic compounds in salt stress response and their effects in prolonged salt stress are still unclear and require further investigation.

## 4. Materials and Methods

### 4.1. Plant Material and Experimental Conditions

Seeds of Chinese cabbage (*Brassica rapa* L. ssp. *pekinensis* (Lour.) Hanelt cv. Cantonner Witkrop) were obtained from ISP, International Seed Processing GmbH, Quedlinburg, Germany. Plants were hydroponically cultivated as previously described [7] at 21 °C, with 16/8 h light (115 µmol m^−2^ s^−1^)/dark cycles. After 4 weeks of cultivation, plant sets were foliar treated (by spraying) with phenolic acids: salicylic acid (SA) and *trans*-ferulic acid (FA) in the concentration range of 10, 50, and 100 µM, and a set sprayed with distilled water. The dose of SA and FA was approximately 0.5 mL per plant per treatment. Half of these pretreated plants were subjected to salt stress, and the NaCl concentration in the hydroponic solution was gradually increased up to 150 mM NaCl. The treatments were started 24 h before salt application and continued for the next 3 days (once daily at the same time). Plants of the same developmental stage that were not exposed to NaCl stress served as controls (C). We examined 8 sets of plants (each set contained 8 plants in a single pot) for each phenolic acid treatment: control (C and foliar spray with water, no NaCl treatment), C + 10 μM FA (SA), C + 50 μM FA (SA), C + 100 μM FA (SA), salt (150 mM NaCl and foliar spray with water), salt +10 μM FA (SA), salt + 50 μM FA (SA), and salt + 100 μM FA (SA). 

The photosynthetic parameters were measured 72 h after salt application in vivo, and plants were harvested, frozen in liquid nitrogen, and stored at −80 °C. The plant material was lyophilized and used for the analysis of stress markers and specialized metabolites.

### 4.2. Fast Chlorophyll a Fluorescence Kinetics

The photosynthetic measurements were performed in vivo with six individual plants per treatment and appropriate controls. Photosynthetic efficiency was determined by chlorophyll *a* fluorescence measurements using a Plant Efficiency Analyzer (PEA, Hansatech, King’s Lynn, Norfolk, UK). The plants were dark-adapted for approximately 30 min before measurements. Chlorophyll fluorescence transients (OJIP) were induced by applying a pulse of saturating red light with a maximum intensity of 650 nm and a photon flux of 3200 µmol m^−2^ s^−1^. Fluorescence changes were measured over 1 s, and the data obtained were analyzed using the JIP-test that represents the translation of the original data into biophysical parameters [53]. To evaluate the state of the photosynthetic apparatus of measured plants, selected structural and functional parameters calculated from the JIP test were selected. The calculations and descriptions of the OJIP test parameters used are shown in Table S1. OJIP transients are presented as mean values of six measurements for each plant group. To compare recorded OJIP transients for specific events in the OP, JP, and IP phases, the difference in relative variable fluorescence was calculated and presented as the differences ΔVOP, ΔVJP, and ΔVIP normalized to the control [18,20].

### 4.3. Proline Analysis

Proline concentrations were determined as described previously for *Brassica* plants [17]. The extraction was performed with 30 mg of the lyophilized tissue in 70% ethanol, and proline concentration was measured at 530 nm with a UV/VIS spectrophotometer (BioSpec-1601 E, Shimadzu, Kyoto, Japan) using a reaction mixture (1% ninhydrin in 60% acetic acid and 20% ethanol). The results are calculated as µmol L-proline per g dry weight (µmol g^−1^ dw).

### 4.4. Determination of Phenolic Compounds and Antioxidant Activity

For the measurement of polyphenolic compounds (total phenolics (TP), total phenolic acids (TPA), total flavonoids (TF), and total flavanols (TFL)) and antioxidant activities (FRAP and DPPH assays), 30 mg of freeze-dried tissue were extracted in 1 mL 80% methanol using a Mixer Mill MM 400 (Retsch, Haan, Germany) for 5 min at 30 Hz, after which the extracts were incubated in a sonicator (10 min) and further mixed in a tube rotator (1 h, 15 rpm). The extracts were then centrifuged (Eppendorf centrifuge, 10 min, 13,000 rpm), and the supernatants were used for all of the analyses described below.

TP were analyzed using the Folin–Ciocalteu method according to Singleton and Rossi [54], and the results were presented as equivalents of gallic acid per dry weight (mg GAE g^−1^ dw). TPA were determined using Arnow’s reagent according to the European Pharmacopoeia [55]; TPA data were expressed as equivalents of caffeic acid per dry weight (mg CAE g^−1^ dw). TF were measured using the AlCl_3_ method [56], and results were presented as equivalents of catechin per dry weight (mg CE g^−1^ dw). TFL were analyzed by the p-dimethylaminocinnamaldehyde (DMACA) method [57], and the results were presented as equivalents of catechin per dry weight (mg CE g^−1^ dw). Antioxidant activity of methanol plant extracts was evaluated by ferric reducing/antioxidant power assay (FRAP) and DPPH radical scavenging capacity assay. The FRAP was measured as described earlier [58]. The results were expressed as μmol Fe^2+^ g^−1^ dw. The DPPH method was measured according to Brand-Williams et al. [59], and the results were expressed as μmol Trolox equivalents per gram dry weight (μmol TE g^−1^ dw). 

### 4.5. Extraction and LC-MS/MS Analysis of Selected Phenolic Compounds

Samples for the analysis of endogenous selected phenolic compounds (salicylic acid, ferulic acid, sinapic acid, kaempferol, and quercetin) were prepared as follows: lyophilized plant material (30 mg) was extracted in 80% MeOH with the addition of the antracene-9-carboxilic acid (ANT) as an internal standard at a final concentration of 20 μg mL^−1^. Supernatants were evaporated in a vacuum concentrator (Sarvant SPD1010 Intergrated SpeedVac System, Thermo Fisher Scientific, Göteborg - Sweden at 40 °C. Pellets were subjected to hot alkaline hydrolysis as described earlier [60]. After adjustment of the pH of samples to 2–3, ethyl acetate was added (3 × 500 μL), the organic phase containing phenolic compounds was collected, and the solvents were evaporated. Dry residues were stored at −20 °C prior to analysis. 

HPLC-grade standard sinapic acid (SiA), *trans*-ferulic acid (*t*-FA), quercetine (QUE), and salicylic acid (SA) were purchased from Sigma Aldrich (Saint Louis, MO, USA), kaempferol (KAE) from Carl Roth (Karlsruhe, Germany), and the internal standard antracene-9-carboxilic acid (ANT) from Alfa Aesar (Haverhill, MA, USA). MiliQ^®^ water (18.2 MΩ cm^−1^; purified by MiliQ water purification system (Millipore, Bedford, MA, USA)) and HPLC gradient-grade methanol (MeOH) (J.T.Baker, Center Valley, USA) were used with analytical-grade formic acid (Acros Organics, Geel, Belgium) for mobile phase preparation. 

The analyte stock solutions (1 mg mL^−1^) in methanol were used for the preparation of calibration curves. Five calibration points were constructed in 50% MeOH + 0.1% formic acid (mobile phase) with the addition of antracene-9-carboxilic acid at a final concentration of 20 μg mL^−1^. Analyte concentrations were: 30–300 μg mL^−1^ for SiA; 6–60 μg mL^−1^ for *trans*-FA, 0.3–1.25 μg mL^−1^ for SA; and 0.02–0.2 μg mL^−1^ for KAE and QUE, respectively. Samples were dissolved in 400 μL of the mobile phase prior to analysis and together with calibrants injected into the LC column at a volume of 20 µL. The calibration curve was obtained by linear regression; the peak area ratio was plotted versus the analyte concentration. A quality control (QC) sample containing 100 ng mL^−1^ of each analyte and instrumental blank were injected after every few runs. During analysis, all instrumental blank samples were negative, and the area of each analyte in QC samples was repeatable. LC-MS/MS analysis was carried out using an Agilent Technologies 1200 series HPLC system equipped with a binary pump, a vacuum membrane degasser, an automated autosampler, and an injector interfaced with 6420 triple quadrupole mass spectrometer with electrospray ionization source (ESI) (Agilent Technologies Inc., Palo Alto, CA, USA). The separation was performed on a Zorbax XDP C18 column (75 × 4.6 mm, 3.5 μm particle size) (Agilent Technologies Inc., Palo Alto, CA, USA). Solvents for the analysis were 0.1% formic acid in water (solvent A) and methanol (solvent B). 

The gradient was applied as follows: 0 min 60% A, 3–12 min 60% A–30% A, 5–20 min 30% A–0% A, 20–25 min 0% A, 25.1–30 min 60% A. The flow rate was 0.3 mL min^−1^. 

The electrospray ionization source was operated in negative mode, and the samples were detected in the multiple reaction monitoring (MRM) mode with a dwell time of 10 ms per MRM transition. The desolvation gas temperature was 350 °C with a flow rate of 6.0 L min^−1^. The capillary voltage was 3.5 kV. The collision gas was nitrogen. Fragmentor voltages were 100 V for SiA, 135 V for KAE and QUE, and 70 V for *trans*-FA, ANT, and SA. The collision energy was set at 15 V for QUE and SA, at 20 V for KAE, at 10 V for SiA and *trans*-FA, and for ANT it was set at 5 V. 

All data acquisition and processing were performed using Agilent MassHunter software.

### 4.6. Lipid Peroxidation Measurement

Lipid peroxidation was determined by the malonaldehyde (MDA) method according to Heath and Packer [61] with a slight modification. Approximately 30 mg of lyophilized plant leaf tissue was homogenized by Mixer Mill Retsch MM 400 for 3.5 min at 30 Hz. To the homogenized powder, 1 mL of 0.1% TCA was added, and the suspension was centrifuged at 13,000 rpm at 4 °C for 10 min. The reaction mixture of either 400 µL of the obtained supernatant (or 400 µL of 0.1% TCA) and MDA reagent (0.5% thiobarbituric acid (TBA) in 20% TCA, Sigma) was incubated at 95 °C for 25 min and centrifuged at 13,000 rpm and 4 °C for 10 min. The absorbance of supernatants was measured at 532 and 600 nm, and the content of MDA was calculated using an extinction coefficient of 155 mM^−1^ cm^−1^. MDA content was expressed as nmol MDA g^−1^ DW.

### 4.7. Statistical Analysis

The data were analyzed with the STATISTICA program (Version Stat Soft. Statistica.v 10.0. Enterprise). ANOVA was used to analyze the relevant factors, and values were considered to be significant at *p* < 0.05. Tukey’s HSD post-hoc test was used for multiple comparisons. Data presented in the text, figures, and tables are means ± standard deviation of three biological replicates (n = 3) for biochemical markers and phenolic compounds. One biological replicate consisted of 7–8 plants grown in the same pot. Fluorescence measurements were performed in vivo, and data are means ± standard deviation of 6 biological replicates (n = 6, one plant is one biological replicate). 

## Figures and Tables

**Table 1:** Formulas and definitions of JIP test parameters [19,20].

Recorded Parameters	Description
F_0_	Minimal fluorescence intensity (20 μs)
F_m_	Maximal fluorescence intensity
V_J_ = (F_J_ − F_0_)/(F_m_ − F_0_)	Relative variable fluorescence at 2 ms
V_I_ = (F_I_ − F_0_)/(F_m_ − F_0_)	Relative variable fluorescence at 30 ms
F_V_ = F_m_ − F_0_	Maximal variable fluorescence
M_0_ = (ΔV/Δt)_0_	Approximated initial slope of relative variable fluorescence F_v_
φ_P0_ = TR_0_/ABS = TR_0_/ABS	Maximum quantum yield of PSII
ψ_E0_ = ET_0_/TR_0_ = 1 − V_J_	Probability that trapped exciton moves an electron further than Q_A_^−^
φ_E0_ = ET_0_/ABS = [1 − (F_0_/F_m_)](1 − V_J_)	Quantum yield for electron transport
δ_R0_ = RE_0_ − ET_0_ = (1 − V_I_)/(1 − V_J_)	Probability with which an electron from the intersystem electron carriers moves to reduce end electron acceptors at the PSI acceptor side
φ_R0_ = RE_0_/ABS = [1 − (F_0_/F_m_)]ψ_E0_ δ_R0_	Quantum yield for reduction of end electron acceptors at the PSI acceptor side
RC/CS_0_ = (ABS/CS)/(ABS/RC)	Density of active RC per excited cross section
ABS/RC = M_0_(1/V_J_)[1/φ_P0_]	Absorption flux per active RC
TR_0_/RC = M_0_(1/V_J_)	Trapping flux per active RC
ET_0_/RC = M_0_(1/V_J_)(1 − V_J_)	Electron transport flux per active RC
DI_0_/RC = (ABS/RC) − (TR_0_/RC)	Dissipation flux per active RC
RE_0_/RC = M_0_(1/V_J_)ψ_E0_ δ_R0_	Electron flux reducing end electron acceptors at the PSI acceptor side, per RC
γRC = Chl_RC_/Chl_total_ = RC/(ABS + RC)	Probability that a PSII Chl molecule functions as RC
PI_ABS_ = [γ_RC_/(1 − γ_RC_)][φ_P0_/(1 − φ_P0_)][ψ_E0_/(1 − ψ_E0_)]	Performance index on absorption basis
PI_total_ = PI_ABS_[φ_R0_/(1 − φ_R0_)]	Performance index for energy conservation from exciton to the reduction of PSI end acceptors

**Table 2:** Content of groups of polyphenolic compounds and antioxidant activities in the methanol extracts of Chinese cabbage leaves subjected to salt stress (150 mM NaCl) and different concentrations of ferulic acid (0, 10, 50, and 100 µM) compared to the untreated control. The results are mean ± SD (n = 3), light gray represents the lowest values, while bright green represents the highest values. Different letters represent statistically significant differences for each individual specialized metabolite compared to treatments (*p* < 0.05). Abbreviations: S, salt (150 mM NaCl); C, control, FA, ferulic acid; GAE, equivalents of gallic acid; CAE, equivalents of caffeic acid; CE, equivalents of catechin; FRAP, ferric reducing/antioxidant power assay; DPPH, 2,2-Diphenyl-1-picrylhydrazyl assay; TE, Trolox equivalents; DW, dry weight.

	Groups of Specialized Metabolites				Antioxidant Activity	
Treatment	Total Polyphenols	Total Phenolic Acids	Total Flavonoids	Total Flavanols	DPPH Assay	FRAP Assay
	mg GAE g^− 1^ DW (%)	mg CAE g^−1^ DW (%)	mg CE g^−1^ DW (%)	µg CE g^−1^ DW (%)	µmol TE g^−1^ DW (%)	µmol Fe^2+^ g^−1^ DW (%)
C	32.66 ± 0.40 c (100)	6.91 ± 0.08 a (100)	5.63 ± 0.03 a (100)	66.46 ± 0.19 bc (100)	41.76 ± 0.60 b (100)	41.07 ± 0.10 b (100)
C + 10 µM FA	35.03 ± 0.20 a (107.26)	6.01 ± 0.02 b (86.98)	5.69 ± 0.04 a (101.07)	75.57 ± 0.79 a (113.71)	40.23 ± 0.17 c (96.34)	44.73 ± 0.54 a (108.91)
C + 50 µM FA	25.52 ± 0.04 d (78.14)	4.10 ± 0.04 g (59.33)	4.12 ± 0.01 d (73.18)	65.38 ± 0.55 c (98.37)	34.66 ± 0.23 f (83.00)	32.42 ± 0.21 e (78.94)
C + 100 µM FA	32.45 ± 0.11 c (99.36)	5.36 ± 0.03 c (77.57)	5.24 ± 0.04 b (93.07)	58.27 ± 0.93 d (87.68)	37.00 ± 0.23 e (88.60)	37.02 ± 0.56 d (90.14)
S	23.57 ± 0.04 e (72.17)	4.32 ± 0.01 f (62.52)	3.81 ± 0.01 f (67.67)	51.02 ± 0.67 f (76.77)	38.98 ± 0.06 d (93.34)	25.99 ± 0.18 fg (63.28)
S + 10 µM FA	34.22 ± 0.12 b (104.78)	5.25 ± 0.03 c (75.98)	4.85 ± 0.01 c (86.15)	67.97 ± 0.93 b (102.27)	51.39 ± 0.23 a (123.06)	39.74 ± 0.46 c (96.76)
S + 50 µM FA	23.60 ± 0.18 e (72.26)	4.84 ± 0.04 d (70.04)	3.71 ± 0.03 g (65.90)	44.04 ± 0.57 g (66.27)	39.85 ± 0.13 cd (95.43)	25.27 ± 0.29 g (61.53)
S + 100 µM FA	24.07 ± 0.13 e (73.70)	4.52 ± 0.03 e (65.41)	3.97 ± 0.03 e (70.52)	53.08 ± 0.54 e (79.87)	37.75 ± 0.50 e (90.40)	26.76 ± 0.07 f (65.16)

**Table 3:** Content of groups of polyphenolic compounds and antioxidant activities in the methanol extracts of Chinese cabbage leaves subjected to salt stress (150 mM NaCl) and different concentrations of salicylic acid (0, 10, 50, and 100 µM) compared to the untreated control. The results are mean ± SD (n = 3), light gray represents the lowest values, while bright green represents the highest values. Different letters represent statistically significant differences for each individual specialized metabolite compared to each treatment (*p* < 0.05). Abbreviations: S, salt (150 mM NaCl); C, control, SA, salicylic acid; GAE, equivalents of gallic acid; CAE, equivalents of caffeic acid; CE, equivalents of catechin; FRAP, ferric reducing/antioxidant power assay; DPPH, 2,2-Diphenyl-1-picrylhydrazyl assay; TE, Trolox equivalents; DW, dry weight.

	Groups of Specialized Metabolites				Antioxidant Activity	
Treatment	Total Polyphenols	Total Phenolic Acids	Total Flavonoids	Total Flavanols	DPPH Assay	FRAP Assay
	mg GAE g^−1^ DW (%)	mg CAE g^−1^ DW (%)	mg CE g^−1^ DW (%)	µg CE g^−1^ DW (%)	µmol TE g^−1^ DW (%)	µmol Fe^2+^ g^−1^ DW (%)
C	27.48 ± 0.17 b (100)	4.89 ± 0.02 d (100)	7.88 ± 0.04 c (100)	58.47 ± 0.58 b (100)	31.92 ± 0.47 b (100)	56.98 ± 0.15 c (100)
C + 10 µM SA	29.79 ± 0.21 a (108.40)	5.81 ± 0.06 c (118.81)	8.84 ± 0.09 b (112.18)	69.07 ± 0.86 a (118.13)	31.56 ± 0.35 b (98.87)	58.40 ± 0.61 b (102.49)
C + 50 µM SA	25.78 ± 0.25 c (93.81)	6.16 ± 0.06 b (125.97)	8.94 ± 0.12 b (113.45)	58.88 ± 1.11 b (100.7)	28.63 ± 0.58 c (89.69)	57.13 ± 0.20 c (100.26)
C + 100 µM SA	30.10 ± 0.24 a (109.53)	6.47 ± 0.05 a (132.31)	9.59 ± 0.04 a (121.70)	43.30 ± 1.14 d (74.06)	35.31 ± 0.57 a (110.62)	62.29 ± 0.28 a (109.32)
S	22.43 ± 0.15 e (81.62)	3.99 ± 0.07 g (81.60)	5.74 ± 0.06 g (72.84)	31.69 ± 0.59 f (54.20)	23.21 ± 0.44 e (72.71)	32.84 ± 0.02 g (57.63)
S + 10 µM SA	21.64 ± 0.22 f (78.75)	4.23 ± 0.03 f (86.50)	7.16 ± 0.07 d (90.86)	34.91 ± 0.77 e (59.71)	23.55 ± 0.34 de (73.78)	35.73 ± 0.40 f (62.70)
S + 50 µM SA	24.79 ± 0.26 d (90.21)	3.80 ± 0.01 h (77.71)	6.10 ± 0.03 f (77.41)	46.93 ± 0.30 c (80.26)	24.77 ± 0.41 d (77.60)	42.86 ± 0.09 e (75.22)
S + 100 µM SA	22.70 ± 0.35 e (82.61)	4.54 ± 0.05 e (92.84)	6.66 ± 0.13 e (84.52)	42.16 ± 0.66 d (72.11)	21.58 ± 0.49 f (67.61)	44.43 ± 0.18 d (77.97)

## Data Availability

Not applicable.

## Supplementary Materials

The following are available online at https://www.mdpi.com/article/10.3390/plants10112346/s1, Figure S1: Representative MRM chromatograms of standard mixture (calibrant 1), Figure S2: Representative MRM chromatograms of sample *B. rapa* treated with 10 μM ferulic acid under salinity stress (150 mM NaCl) (Salt + 10 µM FA), Table S1: Original and normalized chlorophyll a fluorescence parameters measured after 72 h of foliar treatments with ferulic acid (FA) and salicylic acid (SA) at a concentration of 10 µM due to salt treatment (150 mM) compared to the control (C), Table S2: List of phenolic compounds and used internal standard (anthracene-9-carboxylic acid) determined by UPLC-MS/MS, and the MRM transitions of the precursor to product ion pairs.
