# Supplementary material for: Ferulic Acid and Salicylic Acid Foliar Treatments Reduce Short-Term Salt Stress in Chinese Cabbage by Increasing Phenolic Compounds Accumulation and Photosynthetic Performance
Source: Plants (Basel). 2021 Oct 29;10(11):2346. doi: 10.3390/plants10112346 (PMC8619474; doi:10.3390/plants10112346)
Supplement: Supplementary file 1 [file plants-10-02346-s001.zip › Suppl. Figs.pdf]

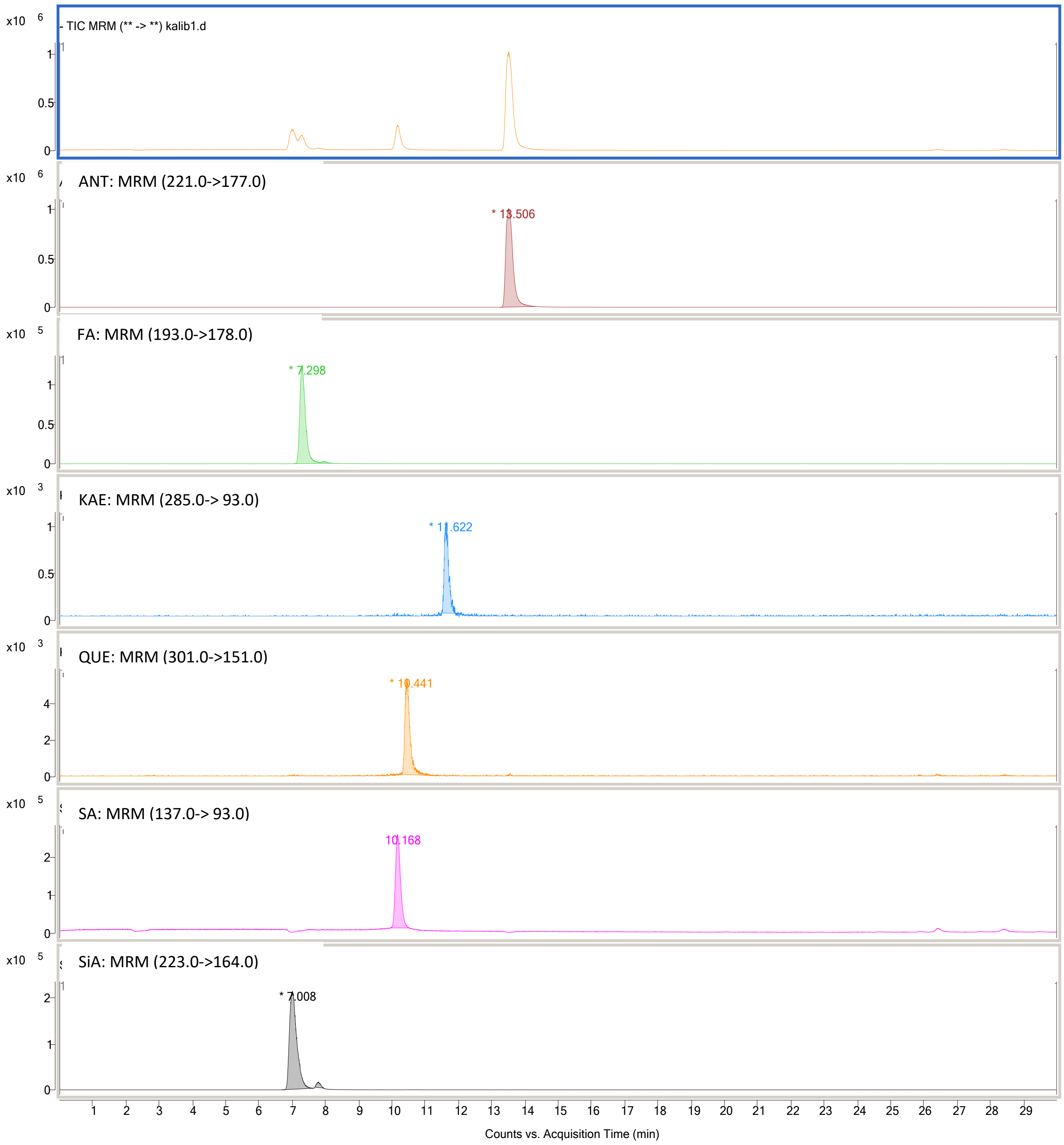

Figure S1. Representative MRM chromatograms of standard mixture (calibrant 1).

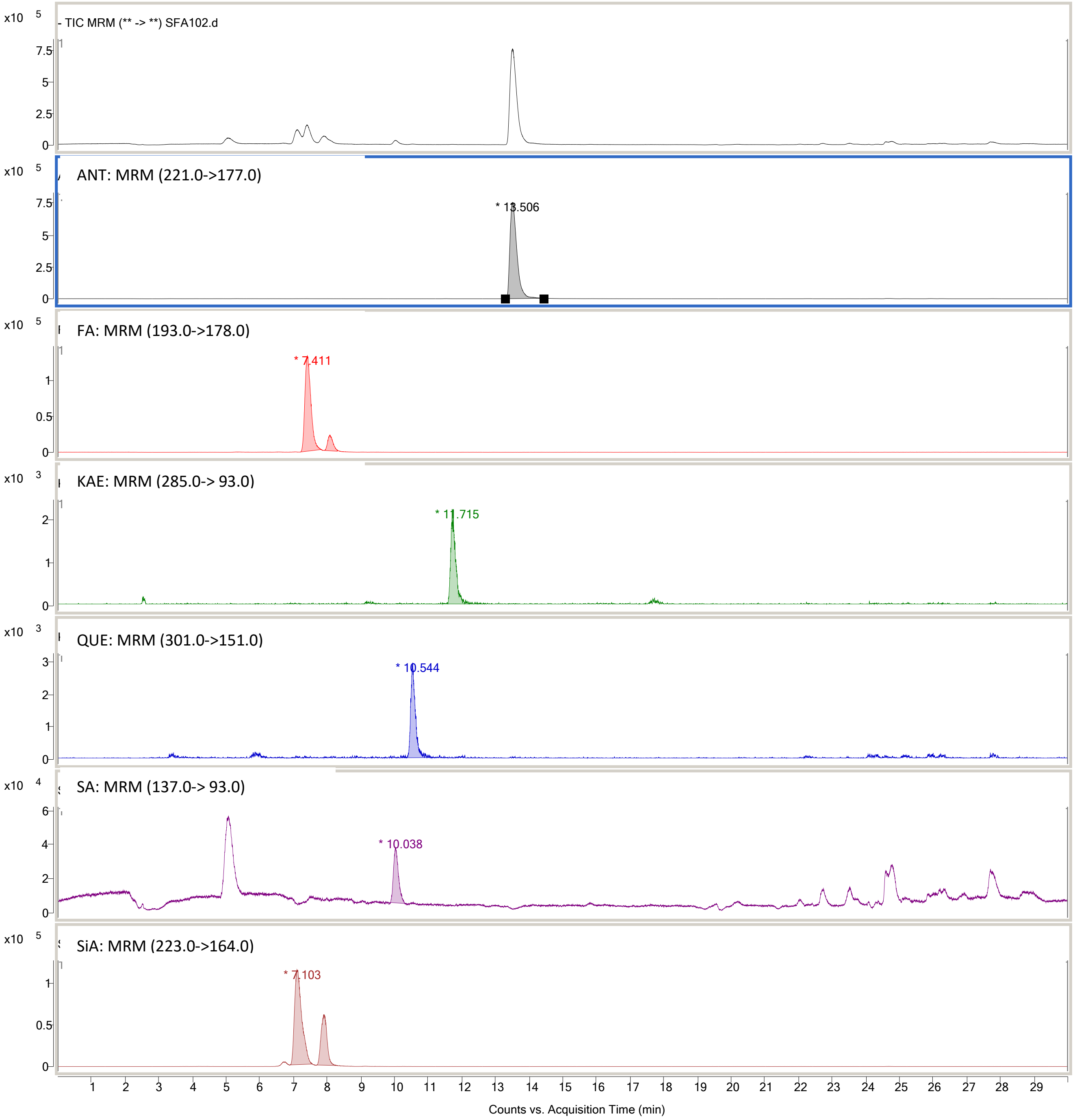

Figure S2. Representative MRM chromatograms of sample *B. rapa* treated with 10 µM ferulic acid under salinity stress (150 mM NaCl) (Salt+10 µM FA). For phenolic acids SiA and FA, in sample chromatograms it was noted that two peaks apear for each analyte. The chromatographic separation of particular isomers is much prefered in lower pH which is nessesarry for adequate sample preparation. This effect was not observed in calibrants prepared in solvents with higher pH values.
